# Blockchain-Based Mobile App for Digital Identification of Older Adults in Rural Peru: Design and Usability Evaluation Study

**DOI:** 10.2196/79553

**Published:** 2026-02-02

**Authors:** Wilver Arana-Ramos, Aldo Francisco Pastrana-Leon, Juan Carlos Morales-Arevalo

**Affiliations:** 1 Department of Software Engineering, Faculty of Engineering, Peruvian University of Applied Sciences, Av. Alonso de Molina Nro. 1611, Lima Polo And Hunt Club (Av. Primavera 2390), Lima, 15023, Peru, +51 994 866 160

**Keywords:** blockchain technology, data privacy, digital divide, essential public service, portable digital identity, older people, user-centric design

## Abstract

**Background:**

Older adults in rural areas of Peru encounter many challenges in accessing critical public services, such as health care, education, and social assistance, due to low levels of digital literacy, limited access to technology, and the lack of formalized, secure ID. This inhibits entry into digital health, education, and social assistance systems and increases their risk of vulnerability and social exclusion.

**Objective:**

This study aimed to design a blockchain technology–based mobile app architecture that helps facilitate secure and inclusive digital ID for older adults in rural areas of Peru, enabling access to vital services through a decentralized, privacy-preserving solution.

**Methods:**

This study followed the design thinking process, which consists of five phases: empathize, define, ideate, prototype, and evaluate. A total of 16 adults (aged 61-85 years) were interviewed to determine the usability barriers and security and privacy concerns with mobile technology, which was used to define functional and nonfunctional requirements. These requirements were developed based on the interviews. The primary features the target population valued included blockchain authentication, assisted registration, multilingual functionality, and a user-friendly interface. The features were prioritized and prototyped using the Figma web-based app. The architecture of the app was developed using the C4 model and accounted for sequential development while ensuring scalability, modularity, and decentralization. Usability was assessed quantitatively by administering the System Usability Scale to the same 16 participants after they had interacted with the prototype.

**Results:**

The mean System Usability Scale score was 60.78 (SD 13.68), indicating acceptable usability. The main issues identified were a lack of skills to navigate digital interfaces, concerns regarding data security, and accessibility challenges for people with disabilities. Participants provided high ratings for the assisted registration system and notifications. The modular, blockchain-based system architecture showed substantial potential for scalability and broader inclusion. The prioritization matrix identified that, for adoption, features must incorporate good design, be multilingual, and require secure authentication.

**Conclusions:**

The proposed blockchain-based mobile app offers a viable technical and socially inclusive model for secure digital ID of older adults in underserved contexts. Usability testing suggested that the solution was perceived as secure, usable, and appropriate for the target population. Although not fully deployed, our prototypes and system architecture provide a good starting point for future implementation. The findings in this study can contribute to efforts to facilitate digital inclusion, access to services, and respect for people’s autonomy in identity management systems for vulnerable people.

## Introduction

Digital technologies have become increasingly important in identity management across all sectors of the economy. The proliferation of mobile apps designed to improve access to services has further deepened this relevance [1]. Older adults living in rural areas of Peru face unique challenges, such as higher levels of digital illiteracy and limited public service coverage. These challenges hinder access to essential services such as health care, education, and social support and mirror broader barriers reported for digital health innovations in low- and middle-income health care systems in South and Southeast Asia [2-4]. Advances in digital identity models, such as self-sovereign identities (SSIs), have opened up new possibilities for improving security, privacy, and access to services [5]. The emergence of identity verification systems that leverage blockchain infrastructure and protocols exemplifies this change, as they ensure there are no gaps in identity verification and authentication processes and allow users to exercise holistic control over their personal information [6].

Nonetheless, traditional identity management systems that typically use centralized data formats and 2-factor authentication often fail older adults in rural settings [7]. Centralized databases are limited, as they rely on dependable connectivity, technical skills, and trust in institutions to function as intended, even though there isn’t adequate infrastructure in many rural contexts. In addition, centralized database models present vulnerabilities by creating a single point of failure that makes any district-based database susceptible to unauthorized access or misuse of information. Furthermore, the use of passwords, tokens, and SMS codes presents usability challenges, as older adults may be less versed in the use of mobile devices and may have difficulty remembering and entering credentials [8]. Conversely, blockchain protocols decentralize validation across identifiable participants and enable user control over personal data by creating a single entry point to the data. Collectively, these properties make blockchain protocols safer, more transparent, and more empowering for this population.

These advances in technology go well beyond data protection. They are fundamental to improving efficiency in the delivery of public and private services. For example, health credential verification and the interoperability of electronic health records are relevant in the health care context. The same is true in education and public participation [9], where the use of mobile apps that integrate SSIs could help reduce the digital divide and provide access to vital resources for all [2,3]. However, their implementation remains limited due to the fragmentation of existing platforms and the lack of a widely accepted interoperable standard. In addition, challenges remain related to centralized data sets, transparency in verification, adherence to strong data privacy policies, and the centralized control of information [10].

In this context, the focus of this study is on designing a blockchain-based mobile app architecture to support digital identity verification and access to necessary services. The goal is to create a system that can be implemented quickly and efficiently while respecting appropriate privacy, security, and decentralization practices in relation to structural data [11].

This study follows a user-centered design thinking (DT) approach to ensure that the proposed solutions are accessible, secure, and appropriate for the target population, rural older adults [12]. The research will include a comprehensive review of current solutions, such as blockchain and smart contracts applied to identity systems, in order to identify the most effective methods for improving user interaction and building trust in digital service platforms [13]. This research is qualitative and will combine a detailed literature review with the design and creation of system architecture models that will eventually be applied in the real world. The goal will be to establish a robust framework for mobile services that use advanced identity verification technologies, highlighting the potential of blockchain to ensure data permanence and integrity. The results of this study provide useful approaches for building mobile systems that prioritize accessibility, security, and transparency, with a special focus on improving essential services [13]. This strategy is even more significant for rural communities, as it allows older adults in these communities to access digital ID solutions that respect their privacy and give them control of their personal information.

Therefore, the objective of this study was to design and evaluate the usability of a blockchain-based mobile app architecture that supports secure digital ID and access to essential services for older adults living in rural areas of Peru.

## Methods

### Ethical Considerations

This research involved 16 older adults (aged 61-85 years) living in rural areas of Peru who voluntarily took part in interviews and usability tests. All participants signed an informed consent form. No sensitive data were collected, and personal information was anonymized and managed in accordance with the principles of confidentiality and data protection.

Formal ethics board (IRB) approval was not required according to institutional guidelines [14], as the study focused on the design and usability evaluation of a digital prototype. The research was non-interventional in nature, involved voluntary adult participants, and did not include clinical procedures or the collection of sensitive personal or health data. All participants provided informed consent prior to participation. The study was conducted in accordance with the principles of the Declaration of Helsinki.

### Research Methodology

This research adopted the DT methodology to develop a mobile app. The user-centered design approach is highly effective in digital product development because it supports users’ needs at every stage while also ensuring a product is functional and accessible to the target audience [15-18]. Among the several established design frameworks, such as user-centered design, participatory design, and agile user experience, DT was selected because it provides a structured yet flexible process that begins with empathy as its core principle. This characteristic aligns with the study’s focus on understanding the lived experiences of older adults in rural Peru, whose limited digital literacy and accessibility challenges require solutions grounded in human context rather than purely technical efficiency. By explicitly situating this work within a lineage of research that views empathy as the foundation for inquiry and intervention, the DT framework allows the research team to translate qualitative insights into practical, socially responsive design decisions [15-17].

The design process was broken into five phases: empathize, define, ideate, prototype, and test, and each phase was important for our design of a blockchain-based mobile product for digital ID and safe access to services. A complete overview of these activities is provided below.

### Empathize Phase

A total of 16 older adults aged 61 to 85 years were interviewed to identify some of the key barriers when trying to access digital services or engage with new technologies. The interviews revealed a number of key issues directly related to participants’ comfort levels and hesitancy with digital technology, and their anxiety surrounding cybersecurity, which often impaired their use of multiple digital platforms. These findings illustrate that solutions must be technically feasible, but also usable, secure, and easy to navigate [15].

### Define Phase

The research team identified important findings from the interviews that influenced the project [19]. Based on the interview findings, the research team identified key user priorities, specifically the importance of a user-friendly interface, transparent privacy control, and user management (input). The study also highlighted the need for ID systems that are achievable and meaningful to users (at all digital literacy levels) [17].

### Ideate Phase

At this stage of the research and development process, the team specified some core features of the app, such as authentication methods incorporating blockchain technologies, a security alerts feature, and personalization options for service access that the user sets according to their needs and preferences. The goal was to create a well-integrated solution in which users could access and manage strategic services relevant to them in a secure and efficient manner [18].

### Prototype Phase

Prototypes and low- and medium-fidelity mockups were developed on the Figma platform. The prototypes helped to visualize the interface design of the app and observe users’ interactions with the app’s functions [19]. The technical system architecture was developed in accordance with the C4 model (this study included only “context,” “containers,” and “components,” excluding “code,” as we did not model the code level) to allow the system to be scalable and adaptable within a blockchain structure [20-22].

### Testing Phase

To quantitatively evaluate prototype app usability, the study applied the System Usability Scale (SUS) developed by Brooke [23] in 1996. The SUS is a well-respected instrument used in usability research, due in part to its simplicity and its provision of a straightforward numerical metric of users’ perceptions of system usability [23].

A survey was created using the standard 10-item SUS, in which respondents indicated whether they agreed or disagreed with statements using a 5-point Likert scale from 1 (“strongly disagree”) to 5 (“strongly agree”). The full set of questions used is in Textbox 1.

The launch survey was conducted on 16 older adults that were representative of the targeted users, all of whom had previous interactions with the prototype. The sample size matched the guidelines recommended for initial usability testing using the SUS. Scoring was based on subtracting 1 from the participants’ response for the odd-numbered items and subtracting the even-numbered item responses from 5. The scores were summed and multiplied by 2.5 to create a final score with a range of 0 to 100, with higher scores representing better usability. User feedback was an important part of the process for improving interface accessibility and ease of use [24]. This cyclical, user-centered approach ensures that the app meets technical specifications while being accessible, safe, and easy to use for older adults with various levels of technological capabilities [16,17].

Textbox 1. **Questions on the System Usability Scale.**

1. I would like to use this application frequently.

2. I found the application unnecessarily complex.

3. I found the application easy to use.

4. I would need the help of a person with technical knowledge to be able to use this application.

5. The various functions of the application are well integrated.

6. I found too much inconsistency in the application.

7. I imagine most people could learn to use this application quickly.

8. I found the application cumbersome to use.

9. I felt very confident using this application.

10. I learned to use the application quickly.

## Results

This section elaborates on the results from the various design phases and the process of developing a mobile app designed for digital identity and secure access to vital services based on blockchain technology, which was achieved using the DT approach. This allowed the team to better understand the needs of the targeted end users, older adults, and ensured that the prototype genuinely reflects the expectations and needs of this group. The major findings and responses presented in each phase are described below.

### Empathize Phase: Interview Findings

During the empathize phase, interviews were conducted with 16 older adults, ranging in age from 61 to 85 years, to identify the barriers and difficulties they face when interacting with mobile apps. The most common problems encountered are presented in Textbox 2.

**Figure 1.**
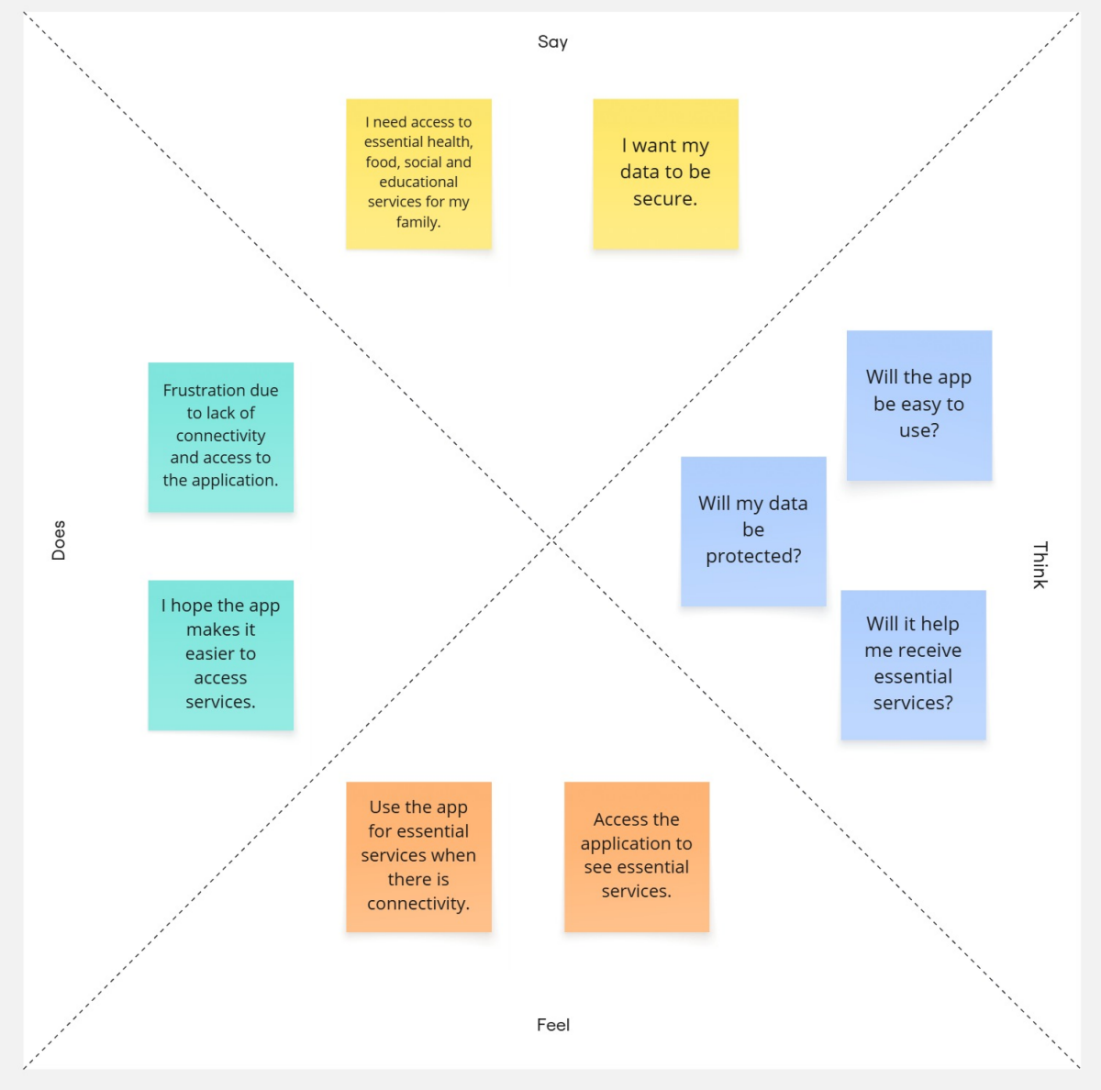
Empathy map: user perspective.

Based on the findings obtained, two empathy maps were developed to reflect the needs and emotions of users in relation to technology [25,26]. These are presented in Figures1  2, where the first corresponds to users and the second to the local authorities.

Textbox 2. **Identified problems.**

- Difficulty navigating apps: users indicated that the app interfaces were complex and difficult to understand.
- Data security concerns: older adults expressed distrust about the handling of their personal data on digital platforms.
- Lack of interface customization: some users reported that the apps were not tailored to their individual needs, making adoption difficult.
- Lack of digital literacy: many older adults have difficulty understanding apps due to their unfamiliarity with digital technologies.
- Limited accessibility: the lack of accessibility options for people with visual or motor disabilities made interaction complicated for a segment of the population.

**Figure 2.**
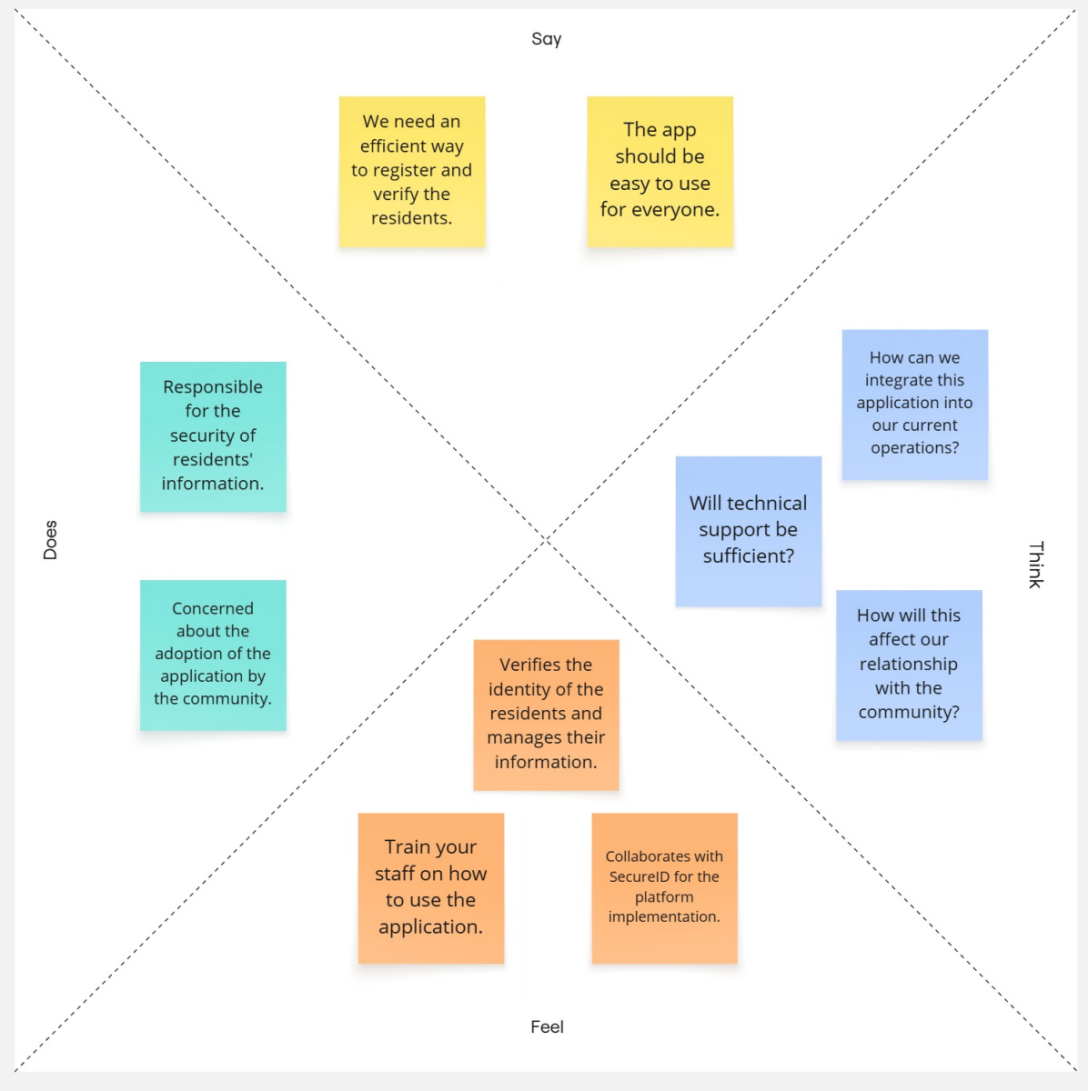
Empathy map: local authorities’ perspective.

### Define Phase: Functional and Nonfunctional Requirements

Based on the findings obtained in the empathize phase, the functional and nonfunctional requirements for the mobile app were defined. These were established based on the specific needs of the users. The most relevant requirements are presented in Table 1 below.

The following are the most relevant user statements, selected from those collected for their significant contribution to understanding users’ expectations regarding interaction with the app and ensuring that their needs are adequately addressed. These user stories are presented in Table 2.

**Table 1.** Functional and nonfunctional requirements.

Requirement	Description	Type
Registration in the app	Allows the user to register in the mobile app with the support of a caregiver, enabling access to their digital identification and essential services.	Functional
Digital identification display	Allows the user to securely access their digital identification based on blockchain technology, guaranteeing the authenticity and protection of their personal data.	Functional
Selection of essential health service	Provides the user with the ability to view and select health services available in their locality, facilitating timely access to vital information.	Functional
Information security	The app must ensure the integrity, confidentiality, and authenticity of user data through the use of blockchain technology and robust security protocols.	Nonfunctional
Multilingual support	The app must support multiple languages to facilitate access to services for users who do not speak the platform’s main language.	Nonfunctional
Notification system	The system must have a notification controller that manages reminders, alerts, and updates, sending them to users and local authorities.	Functional

**Table 2.** Relevant user stories.

User ID	User statement
US-01	As an older adult user, I would like to view my digital identification in order to access my personal data securely.
US-02	As a local authority, I want to filter the list of registered seniors by name or digital identification to quickly locate a specific user and facilitate records management.
US-03	As an older adult user, I would like to register in the application in order to access the available services.
US-04	As an older adult user, I wish to attach and upload images such as a signature, face, or identification to be registered in my account.
US-05	As a local authority, I wish to modify the distribution of essential services.
US-06	As an older adult user, I want to visualize the services provided by the application to easily access the available options.
US-07	As a local authority, I want to view the list of registered seniors to properly track their enrollment and access to available services.
US-08	As a local authority, I want to visualize the services provided by the application to easily access the available options and also have the possibility to add new broadcasts to each selected service.

### Ideate Phase: Key Functionalities and Prioritization Matrix

In this phase, the key functionalities needed for the mobile app were identified. The functionalities were prioritized according to their impact on user experience and the effort required for their implementation. These results are presented in Table 3.

Table 4 shows the prioritization matrix, which ranks the key functionalities according to their impact and implementation effort.

**Table 3.** Selected key functionalities.

Key functionality	Description
Blockchain authentication	A decentralized authentication system to ensure the security and privacy of user data.
Intuitive interface	Simple and accessible design, with customization options to enhance the experience for older adults.
Assisted registration system	Provides personalized assistance during the registration process to ensure the inclusion of older adults who are not fully technologically autonomous.
Multilingual support	The app must support multiple languages to facilitate access to services for users who do not speak the platform’s main language.
Notification system	The system should send notifications to remind users about important events or updates related to services, such as health care, education, and social assistance.

**Table 4.** Functionality prioritization matrix.

Functionality	Impact	Effort	Priority
Blockchain authentication	High	Medium	High
Intuitive Interface	High	High	Medium
Assisted registration system	High	Medium	High
Multilingual support	High	Medium	High
Notification system	High	Medium	High

### Prototyping Phase: C4 Model, User Flow, and Mockups

**Figure 3.**
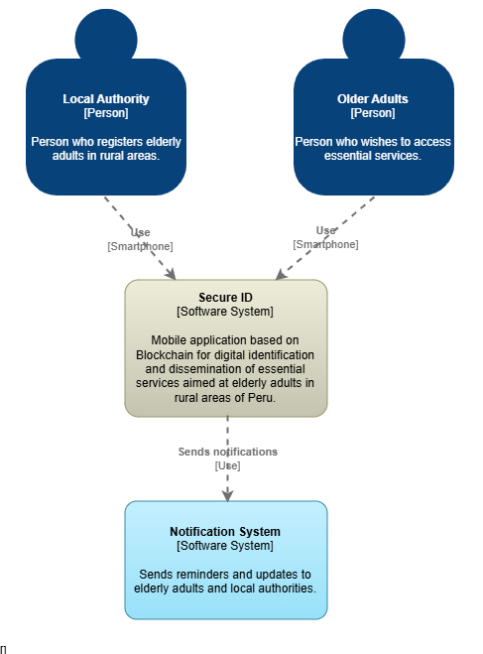
C4 model context diagram.

**Figure 4.**
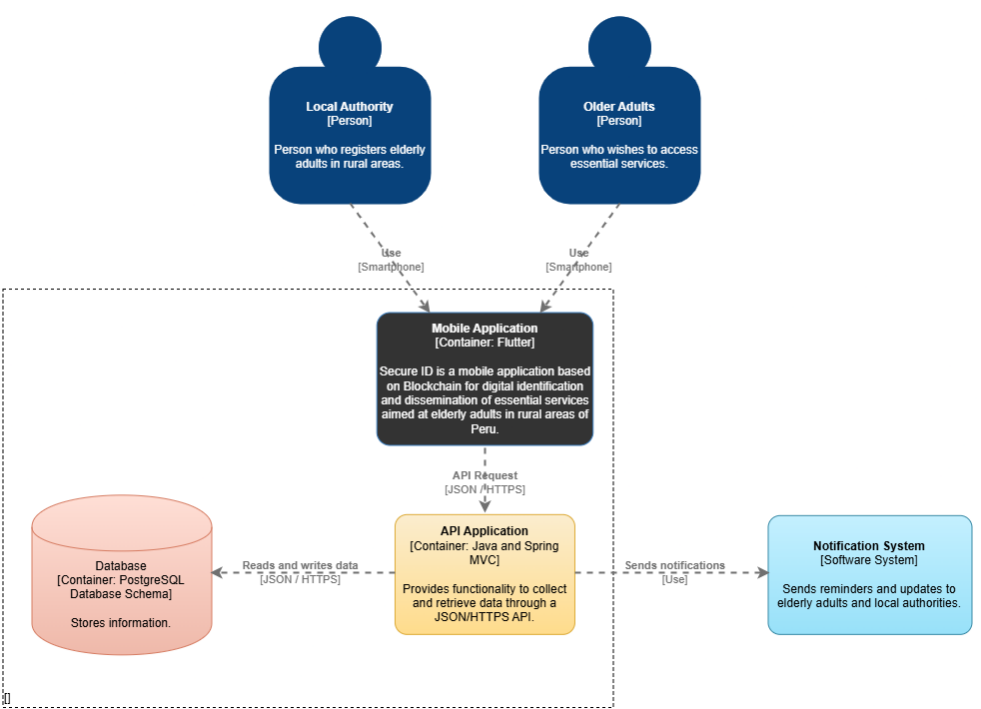
C4 model container diagram.

During the prototyping phase, a comprehensive representation of the system was developed, covering both the technical architecture and the user experience. For this purpose, the C4 model was applied, which allows for structuring the system architecture at different levels of abstraction. Figure 3 shows the context diagram, which illustrates the general interaction between the mobile app, the users (older adults and local authorities), and the external identity verification services. Figure 4 presents the container diagram, which decomposes the system into its main modules: mobile interface, cloud backend, and decentralized database, along with the technologies used. Multimedia Appendix 1 presents the component diagram, which describes the internal elements of the back end, such as the authentication manager, the service controller, and the notification module.

**Figure 5.**
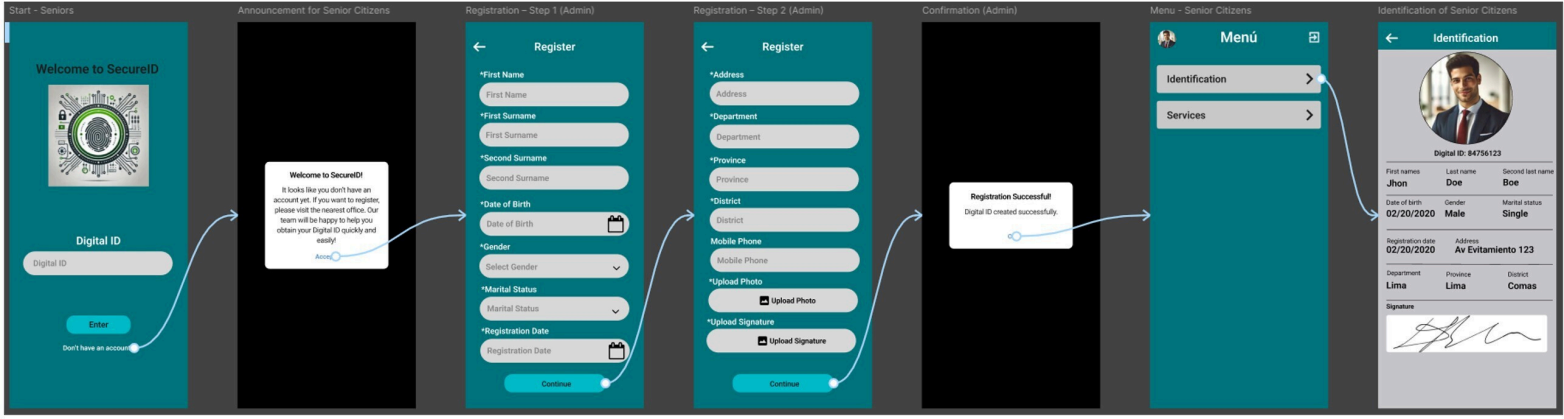
Ideal navigation flow for identification.

To validate the user experience, navigation flows were developed that represent common user steps from login to service access. Figure 5 shows one of these flows, focusing on digital ID.

**Figure 6.**
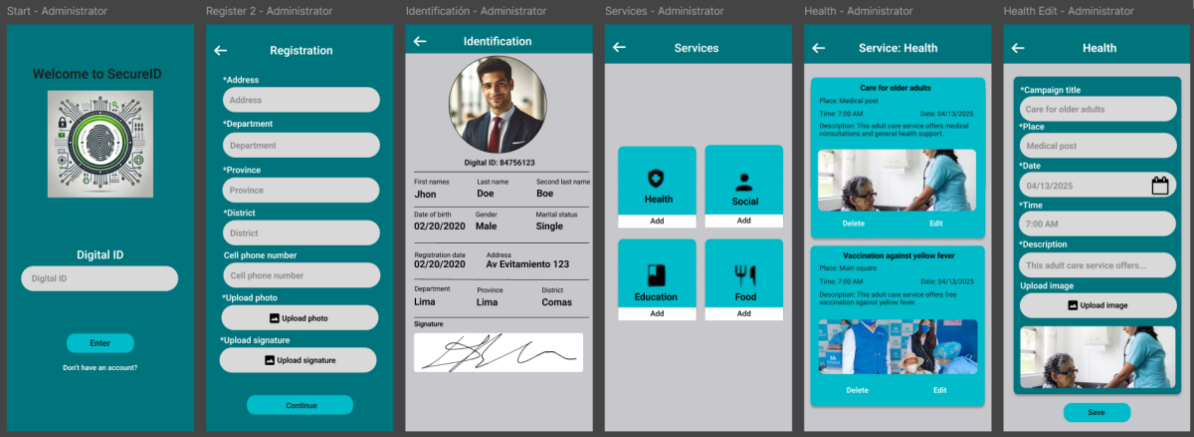
Mockups: registration, identification, and dissemination of essential services.

Finally, low and medium fidelity mockups were designed in Figma. Figure 6 illustrates the most representative screens of the prototype: user registration, ID display, and service dissemination. These prototypes were essential to validate the suitability of the design to the capabilities and preferences of the target audience.

Figure 5 illustrates the proposed navigation flow for the digital ID process of older adults. This sequential design optimizes the user experience through a clear interface and guided steps from the initial registration to the display of the digital ID, ensuring accessibility, usability, and efficiency in identity management.

Figure 6 presents a series of mockups that illustrate key system functionalities from the administrator’s perspective. These include user registration, display of ID information, and management of essential services such as health care, social support, education, and food. The design promotes efficient administration and clear dissemination of campaigns targeting vulnerable populations.

### Testing Phase: Usability Evaluation

**Figure 7.**
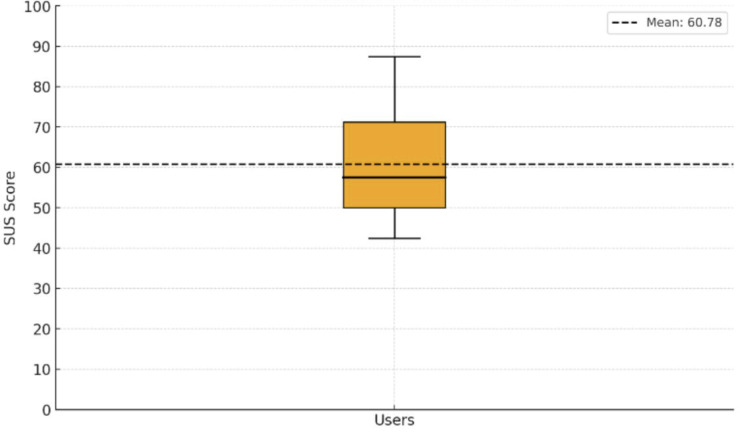
Distribution of System Usability Scale (SUS) scores using a boxplot.

In this phase, the results obtained by applying the SUS to a sample of 16 older adults, representative users of the target population who interacted with the mobile app prototype, were analyzed. The mean SUS score was 60.78 (SD 13.68) on a scale from 0 to 100, where higher values indicate better perceived usability, as can be seen in Figure 7. This score is interpreted as an acceptable usability according to the standards established in the literature, which indicates that the designed prototype is perceived as easy to use, safe, and suitable for the target population.

## Discussion

### Outline

In this study, we designed and evaluated the usability of a blockchain-based mobile app architecture intended to support secure digital ID and access to essential services for older adults in rural Peru. Usability testing with 16 older adults showed that the mean SUS score was 60.78 (SD 13.68), indicating acceptable usability. Older adults viewed the app as secure and mostly easy to use and specifically highlighted the intuitive interface, assisted registration process, and personalized notifications as positive features [25,27], although there were ongoing issues related to digital literacy, trust in data security, and accessibility for users with disabilities.

In terms of accessibility, the design targeted low digital literacy and disability-related barriers through large high-contrast fonts, simplified menus, intuitive iconography, and an assisted registration flow [19]. In our sample, most participants self-reported no visual or motor limitations; those who did generally required more time and occasional assistance during formative sessions, which directly informed these adaptations [8]. The confirmatory evaluation will quantify effectiveness using prespecified metrics, including completion without assistance across core tasks, median task time, error rate, and SUS, stratified by limitation status.

The prototypes and C4 models developed demonstrate a clear and scalable architecture, since they are structured in modular layers that allow incorporating new functionalities without affecting the stability of the system [22]. This scalability is reflected in the use of the C4 model, which facilitates the expansion of the system both in terms of components and cloud services, adapting to different regions or user groups [28]. The solution presented is accessible and reliable, as evidenced by the results obtained in the usability tests with older adults [29].

Unlike the conceptual framework proposed by Tan et al [30], which focuses on a governance taxonomy for blockchain-based systems in the public sector at macro, meso, and micro levels, our proposal is based on a practical and applied approach, which directly implements such principles in a functional architecture with tested prototypes. This framework includes concrete technical decisions on decentralized authentication, user privacy, and accessibility. The designed app facilitates secure and efficient access to essential services, promoting digital inclusion and trust in the handling of personal information [11]. As a limitation, while the study initially focused on design and prototyping, the app is currently being evaluated in real-world settings with older adults from the target population to validate its effectiveness, usability, and adoption in practical environments.

### Related Work

In recent years, academic studies have begun to examine how blockchain technology can reinforce governance structures, support the decentralization of public service delivery, enhance security for mobile apps, and improve the user experience in distributed digital environments. Below are four thematic categories that frame the most relevant work.

#### Application of Blockchain in Public Sector Governance Models

**Figure 8.**
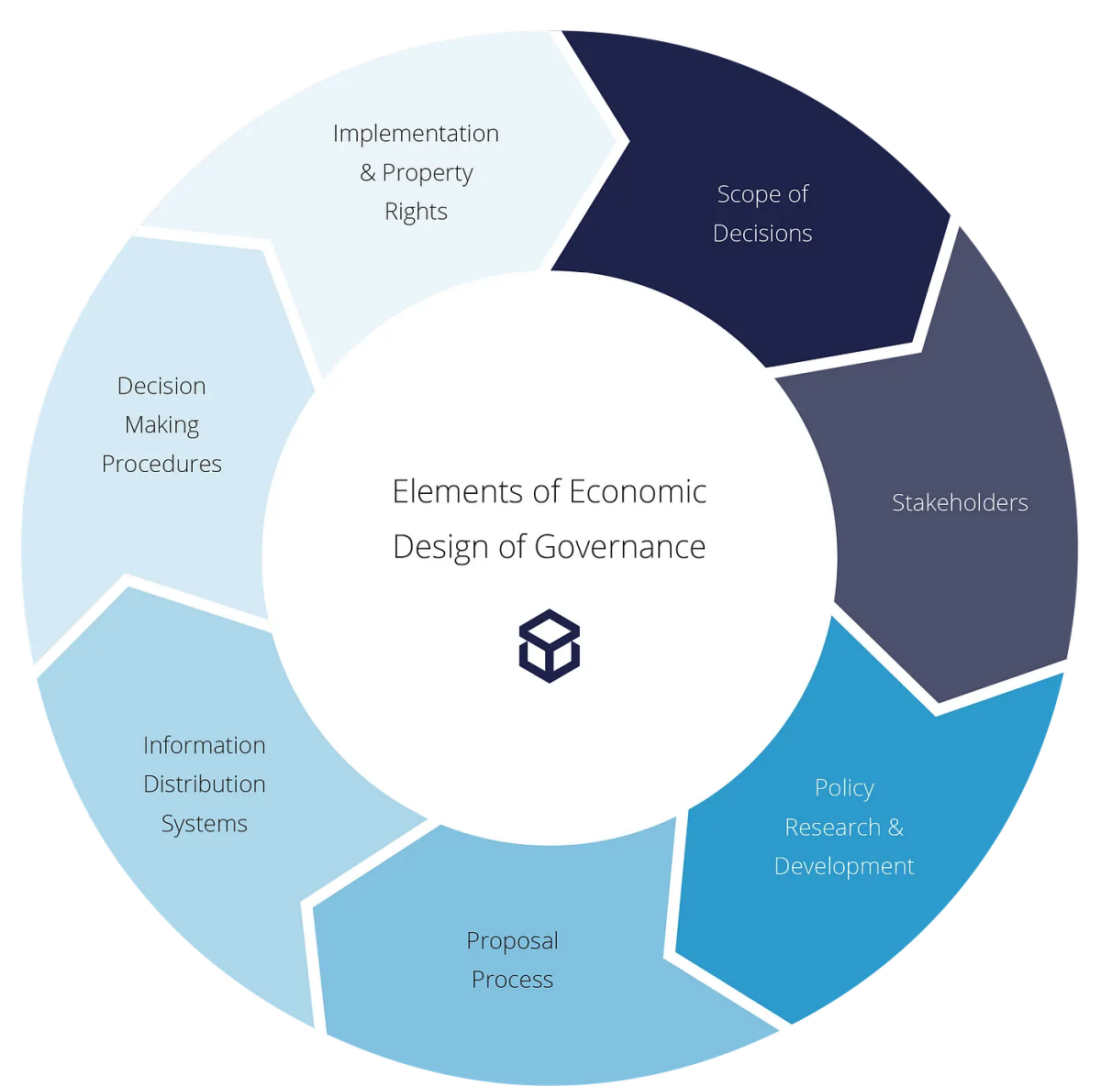
Key elements in the economic design of governance.

Tan et al [30] address the implementation of blockchain’s potential to transform public services by improving transparency, efficiency, and security, key aspects for the design of blockchain-based mobile apps. Through a conceptual framework, the authors explore key governance decisions at three levels: micro, meso, and macro. These governance elements, illustrated in Figure 8, affect the design and implementation of blockchain-based systems in the public sector [31]. However, they identify limitations such as the lack of interoperable infrastructure and the need for effective governance models. This study contributes to these limitations by proposing a solution based on a more flexible approach to digital identity management and access to essential services, especially through mobile apps that enable better interaction with public services [30].

Specifically, the proposed architecture directly operationalizes Tan et al’s [30] microlevel governance principle through user-centered control of digital credentials. For instance, the assisted registration system and blockchain-based authentication module enable users to manage their identity data without relying on centralized authorities. Likewise, the notification and verification features provide transparency and consent management, ensuring that users are aware of and approve any data exchanges. At the mesolevel, interoperability is supported by modular components that facilitate integration with local authority systems, while the macrolevel implications relate to potential scalability within national digital identity strategies. In this way, our design translates Tan et al’s [30] theoretical governance taxonomy into practical app features that promote individual autonomy and trust in digital identity management.

For his part, Ibrahim [32] explores the impact of decentralization on improving public services, highlighting the need to optimize government efficiency and accountability by delegating authority to local governments. This approach, which highlights the importance of tailoring services to local needs, is essential when considering blockchain-based mobile apps for public services. However, Ibrahim notes that disparities in local resources and capabilities limit the effectiveness of decentralization. Our research complements this analysis by integrating blockchain-based technologies, providing a solution that improves security, accessibility, and efficiency in digital ID and access to essential services, overcoming local resource barriers through a decentralized and accessible infrastructure [32].

#### Innovations in Blockchain-Based Mobile Apps for Security and Education

Rizky et al [33] propose a blockchain-based mobile app for decentralization of information management in the field of e-journals, addressing security and reliability issues in centralized systems. Although their focus is on data security in academic platforms, their findings on decentralization and security are highly relevant to the design of mobile apps that manage digital ID in essential public services. Using a SWOT (strengths, weaknesses, opportunities, and threats) analysis and a waterfall development approach, the study implements blockchain to ensure a network resilient to external interference. However, scalability in larger environments remains a challenge. Our research advances this by proposing technical solutions that improve the efficiency and accessibility of essential services, overcoming these limitations by integrating blockchain for secure and decentralized digital ID.

**Figure 9.**
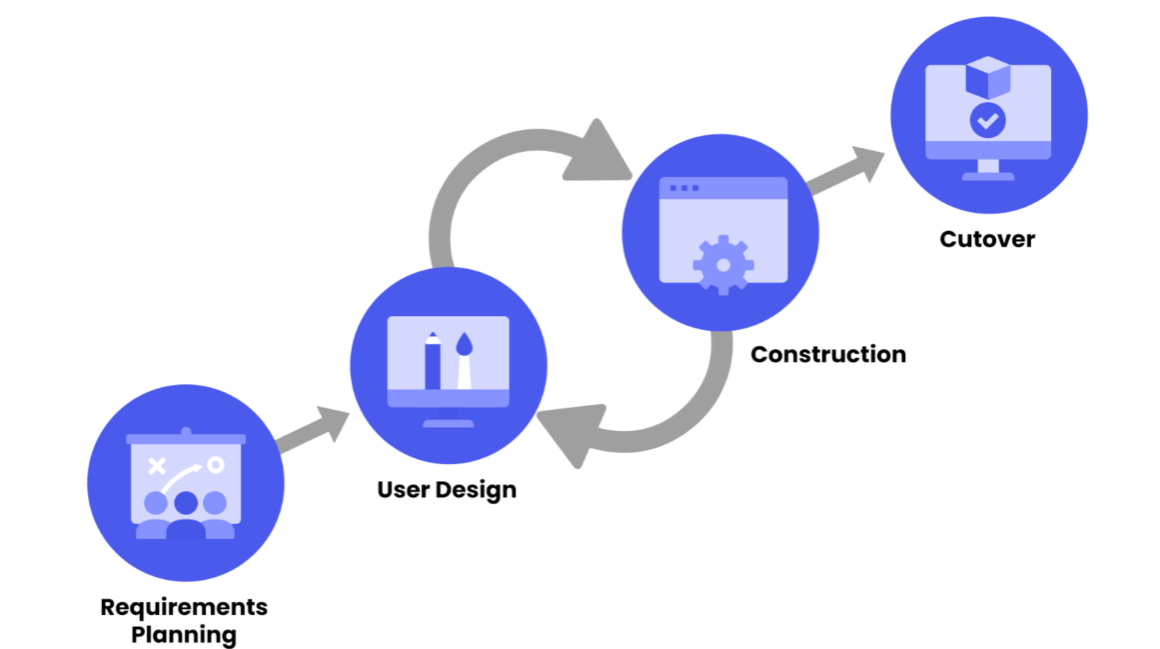
Rapid application development model phases.

Similarly, Asmawi et al [34] developed BlockScholar, a blockchain-based mobile educational app to facilitate the understanding of blockchain. Their research addresses the gap in interactive and accessible educational resources, highlighting the need for platforms that offer immersive and accessible learning on blockchain. They used the rapid application development model to create interactive and gamified content, as illustrated in Figure 9. Although the study made progress in accessibility and comprehension, it still faces challenges in adaptability to diverse educational contexts. Therefore, our research proposes solutions that enhance learning personalization and expand the coverage of blockchain-related topics.

#### Security, Identity, and Digital Inclusion with Blockchain

Gumilar et al [35] explore the integration of financial technology in digital inclusion, with an emphasis on digital financial literacy as a catalyst for reducing economic and social disparities [35]. Through a systematic literature review, they used Scopus databases (2020-2024) to examine how the adoption of digital financial services optimizes inclusion. The results highlighted the importance of improving digital financial literacy but noted limitations in consumer use and protection. In line with these findings, our research extends the analysis by proposing innovative technological solutions and a more accessible design to improve adoption in underserved populations.

On the other hand, Musa et al [36] propose a blockchain-based approach to improve the security of data storage in Android mobile apps. They address the problem of vulnerability and unauthorized access to sensitive data by using blockchain to provide decentralized and secure storage. Their methodology includes the implementation of a 6-layer framework called BSADS (blockchain-based secure android data storage), which optimizes efficiency and security, as detailed in their proposed framework. Despite advances, scalability and costs remain limitations. This research contributes to overcoming these challenges by proposing lightweight node solutions and cost optimization techniques.

#### Identity and User Experience Management on Blockchain

Alanzi and Alkhatib’s [37] study presents blockchain-based identity management solutions aimed at improving privacy and security in traditional centralized systems. Using blockchain technologies such as Ethereum and smart contracts, they address issues of third-party control, single point of failure, and vulnerability to data manipulation. However, limitations in scalability and weak authentication of some proposed systems are identified. In this context, our research extends these approaches, proposing improved decentralized infrastructure and optimizing the design of smart contracts to increase security and efficiency in digital identity management systems.

On the other hand, Jang and Han [38] developed a user experience framework for blockchain-based services, specifically focused on improving user interaction in contexts such as finance and health care [38]. Through an analysis of active services, they identified both general and blockchain-specific functions, highlighting improved efficiency and security. However, their study faces limitations in general applicability due to a lack of standardization. Consequently, our research extends this framework, addressing the implementation of blockchain in digital identity and essential services, improving accessibility and user experience through advanced technological solutions [38]. Table 5 summarizes the key approaches and inputs underlying the development of a blockchain-based mobile app for digital ID and access to essential services.

**Table 5.** Fundamental approaches for blockchain-based mobile apps.

Approach	Contribution	Referenced works
Taxonomy of governance	Rationale for the governance approach	Tan et al [30]
Decentralization	Decentralization analysis	Ibrahim [32]
Patterns of mobile apps	Mobile app design patterns	Rizky et al [33] Asmawi et al [34]
Inclusion, security, identity, and user experience	Design and accessibility considerations Blockchain security and authentication Digital identity management User experience and accessible design	Gumilar et al [35] Musa et al [36] Alanzi and Alkhatib [37] Jang and Han [38]

### Conclusions

This study designed a functional architectural proposal for a blockchain-based mobile app that facilitates digital ID and access to essential services, with a focus on the inclusion and data security of older adults. The solution addresses usability, privacy, and decentralization issues, overcoming the limitations of traditional centralized and vulnerable systems. In addition to its technical and usability benefits, the proposed system entails ethical challenges related to data governance. If local authorities or institutions act as blockchain nodes, they could potentially exert undue control over users’ identity data. To prevent this, future implementations should ensure transparent node management, community oversight, and independent auditing. These measures are key to promoting genuine digital inclusion while safeguarding autonomy and trust in rural contexts. Nevertheless, usability tests applied using the SUS revealed high acceptance and perceived ease of use by older adults, demonstrating that the proposal represents a solid basis for the comprehensive development of the app, which has been implemented in a functional prototype currently undergoing real-world testing with older adult participants in rural areas.

## Supplementary material

10.2196/79553 Multimedia Appendix 1 C4 model component diagram.

## Abbreviations

DT: design thinking
SSI: self-sovereign identity
SUS: System Usability Scale
