# Supplementary figures and images for: Blockchain-Based Mobile App for Digital Identification of Older Adults in Rural Peru: Design and Usability Evaluation Study
Source: JMIR Rehabil Assist Technol. 2026 Feb 2;13:e79553. doi: 10.2196/79553 (PMC12863781; doi:10.2196/79553)

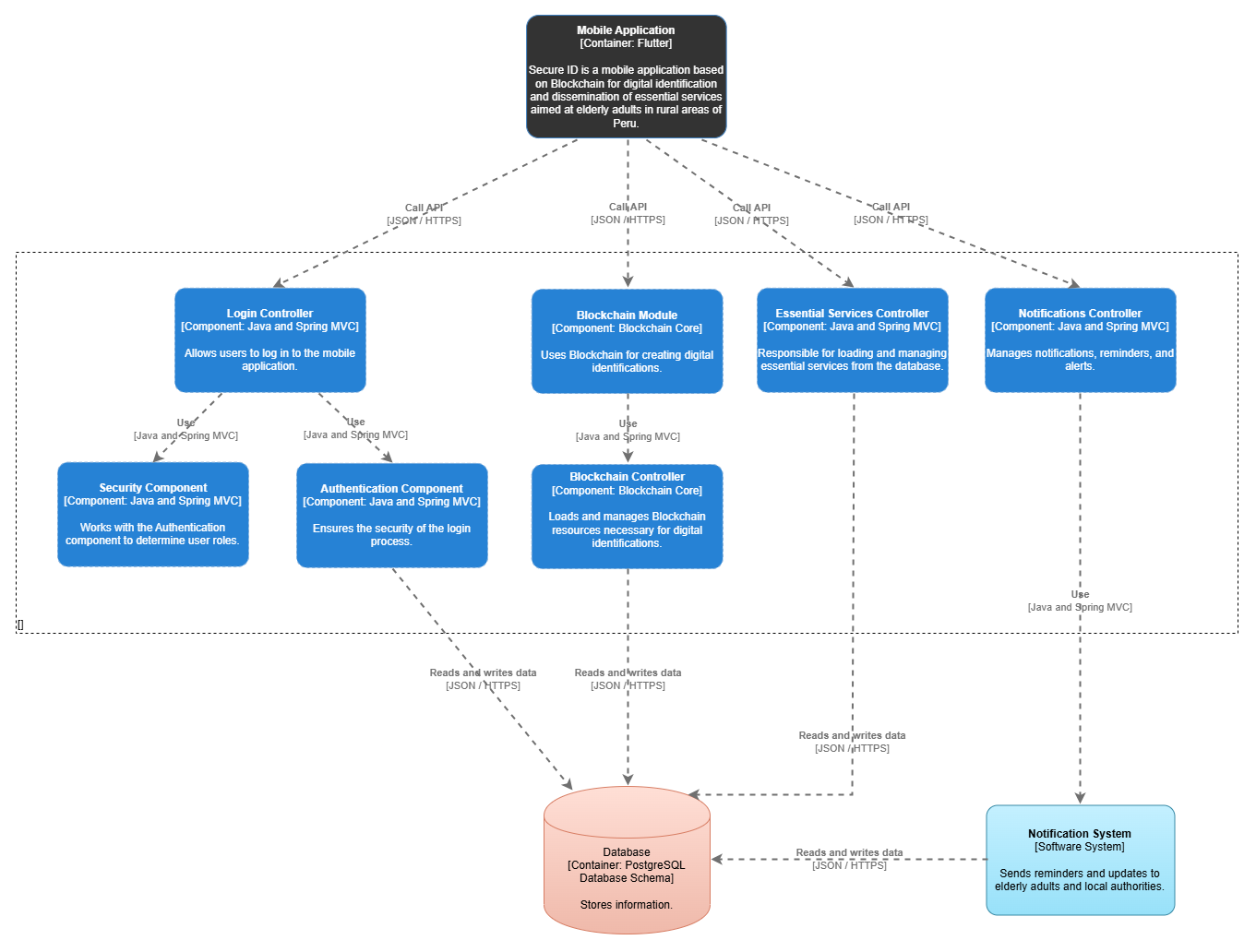

Supplement: Multimedia Appendix 1 [file rehab-v13-e79553-s001.png]
